# Prevalence and factors associated with fertility desire among HIV-positive women in Rwanda in the context of improved life expectancy

**DOI:** 10.1186/s13690-021-00742-w

**Published:** 2021-11-25

**Authors:** François Niragire, Celestin Ndikumana, Marie Gaudence Nyirahabimana, Dieudonne Uwizeye

**Affiliations:** 1 Department of Applied Statistics, University of Rwanda, Kigali, Rwanda; 2 Department of Governance and Public Administration, University of Rwanda, Kigali, Rwanda; 3 CARE International- Rwanda, Kigali, Rwanda; 4 Department of Development Studies, University of Rwanda, Kigali, Rwanda

**Keywords:** HIV/AIDS, Fertility desire, Factors, Logistic regression, Odds ratio, Rwanda

## Abstract

**Background:**

The knowledge of the key factors associated with fertility desire among people living with HIV/AIDS is crucial for the efficient planning of maternal and child health care programs. Fertility desire has generally increased among women of reproductive age in Rwanda. However, its level and determinants among women living with HIV/AIDS (WLHA) are currently not well known in the context of Rwanda. The present study aimed to fill in this knowledge gap.

**Methods:**

Data were extracted from the 2015 Rwanda demographic and health survey (RDHS) for 243 HIV-positive women of reproductive age. Univariate and multivariable logistic regression analyses were conducted in order to identify the most influential factors.

**Results:**

The prevalence of desire to have another child in HIV-positive women was found to be as high as 40.7%. Multivariable logistic regression analyses showed that the woman’s age of 35–49 years (AOR = 0.051, 95% CI: 0.013–0.204), woman’s parity of 3 children or above (AOR = 0.177, 95% CI: 0.037–0.837), being employed (AOR = 0.298, 95% CI: 0.113–0.782) and currently using contraceptives (AOR = 0.146; 95% CI: 0.057–0.375) were significantly associated with low odds of fertility desire among HIV- positive women in Rwanda. Women younger than 25 years, with no living child, or who were unemployed or who were not using any contraceptive were significantly associated with greater odds of desire to have another child than did other HIV- positive women. A woman whose partner's desire for children is different  from hers was associated with about four times higher odds (AOR = 3.752; 95% CI: 1.203–11.702) of desire for more children than women who desire the same as their partners.

**Conclusion:**

Fertility desire in WLHA is currently high in Rwanda. It is significantly influenced by demographic and socioeconomic factors. The Rwanda’s health care system should be prepared to intensify the required services for the prevention of the vertical transmission of HIV, the delivery of maternal and child health care services, and the support to WLHA in planning their fertility. Interventions should target low-parity young women, with a particular focus on meeting their contraceptive needs.

## Background

Globally, around 38.0 million people were living with HIV at the end of 2019. Around 19.2 million (more than 50%) of them were women who were at least 15 years old. The majority of women living with HIV/AIDS (WLHA) (15.9 million) were residing in Sub-Saharan Africa and were of childbearing age [1]. Despite the risks associated with HIV infection for them [2, 3], many of these women still desire more children [4–6]. Adolescent girls and young women accounted for 1 in 4 new infections in Sub-Saharan Africa [7] in 2019. Besides, in eastern and southern Africa, three in five new infections occurred among women, and adolescent girls and young women (aged 15 to 24 years) were 2.5 times more likely than their male peers to acquire HIV infection [7]. In Rwanda, 3.0% of the population aged 15–49 are HIV-positive. HIV infection prevalence is higher in women (3.6%) than in men (2.2%). Among women, HIV prevalence is lowest (0.9%) at age 15–19 and highest (7.8%) at age 40–44 [8]. In 2019, 99% of pregnant women living with HIV were accessing antiretroviral medicines [1].

Undoubtedly, the desire to have more children among people living with HIV/AIDS (PLHA) has implications for women’s health, delivery of health care services as well as the implementation of health policy and programs [9]. It increases the demand for maternal and child health care services including prevention of mother-to-child transmission of HIV. Studies also reported mental disorders, increased abortion and other health problems related to pregnancy in HIV-positive women that are potential to induce depression, self-denial and condemnation [3, 10–12]. Also, HIV-infected pregnant or post-partum women are more likely to die [2]. Furthermore, HIV/AIDS infection contributes to the decrease of fertility among PLHA by causing abortion and stillbirth or through decreased sexual desire and marital disruption among other ways [2, 10, 11].

Thus, HIV infection causes profound changes in the social life, fertility intention, sexual and reproductive behaviour of the affected individuals and community [2, 10]. This is partly the consequence of the economic impact of HIV/AIDS, deterioration of health conditions, and increased likelihood of deaths that have been attached to being HIV-positive [10, 13, 14].

Fortunately, the world has recently witnessed important improvement in life expectancy of HIV/AIDS affected patients mainly due to early initiation and rapid scale-up of antiretroviral therapy in several countries [15–17]. Such changes in health outcomes are usually accompanied by changes in various life dimensions including sexual and reproductive behaviours as well as their determinants [5, 18–20].

Several studies have been conducted to understand the fertility desire and the reproductive health behaviour of people living with HIV/AIDS. These studies have shown that the potential factors associated with fertility intention or desire include the woman’s age [4, 21–23], marital status [5, 22], educational level [5, 24], household wealth or economic status [4], family size, woman’s parity [4, 21–23], fertility desire of the woman’s partner [23, 25], levels of women’s empowerment and gender equity, woman or her partner’s employment status and occupation [26–28]. Further potential factors include contextual factors such as region and urban residence, religious belief, cultural norms and traditions [4, 25, 29]. Being on antiretroviral therapy led to differing results for fertility desire and intention in HIV-positive patients [5, 19, 21, 30].

The literature shows that the key factors associated with fertility desire or intention vary from one community or country to another [1, 4, 21–23, 31]. Therefore, overgeneralising the determinants may not provide adequate information to decision makers and implementers of health programs.

Rwanda is a landlocked East African country of 26,338 km^2^ [8]. Its population was 10,537,222 in 2012 with an annual growth rate of 2.6% [32]. In Rwanda, the national HIV prevalence rate in the population between 15 and 49 years old is estimated at 3%, being higher in women (3.6%) than in men (2.2%) [8]. The total fertility rate in women between 15 and 49 years old declined from 6.1 in 2005 and 4.6 in 2010 to 4.2 in 2015 [8].

Rwanda is one of the countries where the life expectancy of HIV-positive patients improved substantially and became comparable to the general population’s life expectancy [15]. The improvement suggests changes in fertility desire and intention among PLHA [5, 18, 19]. In Rwanda, the proportion of women wanting more children has increased from 44% in 2010 to 49% in 2014–15 [8]. Thus, the assumption of concurrent changes, and possibly increase, in fertility desire among PLHA in Rwanda, specifically HIV-positive women deserves testing [19]. There is a need to understand the determinants of fertility desire among HIV- positive women in Rwanda to inform the planning and provision of reproductive health services including the prevention of mother- to-child transmission (PMTCT) of HIV [33]. The present study aim was to assess fertility desire among HIV- positive women and its determinants in Rwanda.

## Methods

### Study data and sample

This is a quantitative cross-sectional study based on an analysis of data from the 2015 Rwanda demographic and health survey (RDHS). The latter was conducted by the National Institute of Statistics of Rwanda (NISR) between November 2014 and April 2015 [8]. It is a population-based and nationally representative survey in Rwanda that tested for HIV infection and collected numerous characteristics on individual women and men as well as their households. The RDHS data collection personnel were trained by the NISR with support from ICF International in October 2014 [8]. Details about the 2015 RDHS methodology are well documented in the related report [8].

To investigate the factors associated with fertility desire among HIV-positive women in Rwanda, each woman’s HIV test result and her personal and household’s characteristics were matched based on two data files from the 2015 RDHS, namely the individual women’s data (Individual Recode file) and HIV Test data (AIDS Recode file). Thus, the matched-data were suitable for the analysis of the factors associated with fertility among HIV-positive women in Rwanda. In total, 13,497 women were interviewed and an HIV test was done for 6749 women during the 2015 RDHS. Among them, 254 (3.8%) women aged 15–49 years were HIV-seropositive. Six HIV-positive women or their partners were sterilized, 4 were declared infecund and one had missing information on the fertility desire variable. In sum, 11 HIV-positive women were removed from the analysis. Thus, the results of the present study are based on data from a sample of 243 HIV-positive women who had complete data for the analysed variables.

### Study variables

#### Outcome variable

The outcome variable in this study is the woman’s fertility desire to have another child. During the 2015 RDHS, this variable was measured with six values namely, ‘have another’, ‘undecided’, ‘no more’, ‘sterilized’ (respondent or partner), ‘declared infecund’, and ‘never had sex’ [8]. The question was asked regardless of whether the woman was pregnant. In this study the outcome variable was recoded into two levels that indicate whether the woman expressed desire of ‘*no more’* child (coded with 0) or ‘*have another*’ child (coded with 1). Except for the ‘undecided’ persons, respondents who answered with the other values were excluded from the analysis because they cannot be associated with the choice of fertility desire or intention.

#### Explanatory variables

Proximate-determinants conceptual frameworks for fertility analysis [34, 35], and other existing literature [6, 22, 23, 33, 36] guided the selection of potential factors influencing fertility desire among HIV-positive women. There were different health, social, economic, demographic, cultural and proximate (behavioural) variables that were collected during the 2015 RDHS. They include woman respondent’s age, marital status, the highest level of education, occupation, employment status, age at first sex, age at first cohabitation, whether she had a co-wife (polygamy), number of sexual unions, recent sexual activity, contraceptive use, number of living children, the ideal number of children, religion, province of residence, type of place of residence, partner’s desire for children, partner’s age, partner’s occupation, partner’s educational level, and household’s economic status [8]. Often, there are strong relationships between groups of variables such as the sexual and reproductive behaviour variables (age at first sex, age at first cohabitation, number of sexual unions, existence of a co-wife or polygamy, etc.) [37, 38].

In this study, all categorical variables were dummy-coded, and the first category was the reference category. Most variable categories were chosen and recoded based on the existing literature to enable comparisons with other studies [36]. The household’s economic status was derived from the DHS household’s wealth index variable which was reported with five quintiles [39]. The ‘poorer’, ‘middle’ and ‘richer’ quintiles were grouped into a ‘medium*’* economic status, while the ‘poorest’ and ‘richest’ quintiles were recoded into *low* and *high* economic statuses respectively [36]. The ‘ideal number of children’ refers to the number of children to whom a woman would want to give birth in the course of her reproductive life. The woman’s recent sexual activity was considered for the preceding four weeks [8]. For current ‘marital status’ variable, those women who were ‘married’ or ‘living with partners’ were grouped into ‘*living with partner*’ category while those ‘widowed’, ‘divorced’, ‘separated’ or ‘never in union’ (never or no longer in sexual union) were grouped as ‘*not in union*’. For partners’ characteristics, women who were ‘not in union’ formed a separate category. During the 2015 RDHS, information about contraception practice was collected by contraception methods. The latter were classified as folkloric methods, traditional methods, or modern method. The survey also recorded whether the respondent was not using any method [8]. In this study the variable “current contraceptive use”, indicates whether the respondent was using a modern contraception method or not (folkloric, traditional or no method).

### Statistical analysis

In the present study a series of chi-square tests of independence were used to test the association of each variable with the woman’s fertility desire. All variables, including those with test *p*-values greater than 0.05 were considered for the multivariable logistic regression analysis. Data management was carried out using IBM SPSS Statistics for Windows version 20.0.

The outcome variable, the woman’s fertility desire, is binary. Thus, a binary logistic regression model is suitable for the analysis of potential factors’ effects on fertility desire in HIV-positive women [40]. Specifically, if *Y*_*i*_ denotes the outcome variable taking on “have another” child, with an unknown probability *p*_*i*_ or ‘no more’ child with probability 1 − *p*_*i*_, and if the set of covariates *X* contains *k* factors (including all dummy variables), then the logistic regression model for a HIV-positive woman *i* desirous to have another child is given by eq. (1) [40].

1 $$ l\mathrm{ogit}\left({p}_i\right)={\beta}_0+{\beta}_1{x}_{i1}+\cdots +{\beta}_k{x}_{ik} $$

The linear predictor, *η*_*i*_ = *β*_0_ + *β*_1_*x*_*i*1_ + ⋯ + *β*_*k*_*x*_*ik*_, is such that the model parameter vector *β* = (*β*_0_, ⋯*β*_*k*_)^'^ of linear fixed-effects is estimated using the study data. For each parameter *β*_*j*_, *j* = 1, ⋯, *k*, the value exp(*β*_j_) is the relative adjusted odds ratio (AOR) of desire to have another child for a woman with attribute *x*_*j*_ [40]. Interpretation of the present study results were based on estimated adjusted odds ratios and their corresponding 95% confidence intervals (CIs).

## Results

### Characteristics of study subjects

According to the results, 99 (40.7%) of the 243 HIV-positive women expressed a desire to have another child. The results in Table 1 show the frequency distribution of fertility desire among sampled HIV-positive women according to the selected potential factors. This study data show that 51.9% of the sampled women were living with partners, 50.6% had been sexually active in the 4 weeks preceding the survey, 51.4% had experienced sex before turning 19 years, 58.0% had only been in one sexual union, 69.1% reported that they anticipated more than 2 children, and 65.4% said they were not using any contraceptive method by the 2015 RDHS survey interviews. This study also found that the majority of women (63.0%) had completed at most primary education, 80.7% were employed, 53.1% were living in rural areas, and 51.9% were working in agriculture, while 40.7% were from medium- economic status households, and 44.9% were from protestant churches.

**Table 1:** Distribution of woman’s fertility desire and test of its association with potential factors (*n* = 243)

Variable	Categories	Category Total (% of sample)	Desire more child (% of category)	***P***-value
*Woman’s age (years)*	15–24	38 (15.6)	31 (81.6)	< 0.001
	25–34	94 (38.7)	50 (53.2)	
	35–49	111(45.7)	18 (16.2)	
*Marital status*	Living with partner	126 (51.9)	52 (41.3)	0.862
	Not in union	117 (48.1)	47 (40.2)	
*Has a co-wife*	No	108 (44.4)	47 (43.5)	0.431
	Other	135 (55.6)	52 (38.5)	
*Sexually active*	No	123 (50.6)	46 (37.4)	0.283
	Yes	120 (49.4)	53 (42.2)	
*Age at first sex*	Below 19 years	125 (51.4)	49 (39.2)	0.615
	Other	118 (48.6)	50 (42.4)	
*Number of sexual unions*	Once	141 (58.0)	55 (39.0)	0.518
	Other	102 (42.0)	44 (43.1)	
*Ideal number of children*	At most 2	75 (30.9)	35 (46.7)	0.209
	3 or above	168(69.1)	64 (38.1)	
*Current contraceptive use*	No	159(65.4)	83(52.2)	< 0.001
	Yes	84(34.6)	16(19.0)	
*Religion*	Catholic	85 (35.0)	31 (36.5)	0.568
	Protestants	109 (44.9)	48 (44.0)	
	Other	49 (20.2)	20 (40.8)	
*Woman’s education*	No education	45 (18.5)	16 (35.6)	0.153
	At most Primary	153 (63.0)	59 (38.6)	
	Secondary or higher	45 (18.5)	24 (53.3)	
*Number of living children*	None	29 (11.9)	25 (86.2)	< 0.001
	1–2	107 (44.0)	60 (56.1)	
	3 or above	107 (44.0)	14 (13.1)	
*Currently employed*	No	47 (19.3)	28 (59.6)	0.003
	Yes	196 (80.7)	71 (36.2)	
*Partner’s desire for children*	Wants the same	84 (34.6)	30 (35.7)	0.246
	Other	159 (65.4)	69 (43.4)	
*Household’s economic status*	Low	47 (19.3)	14 (29.8)	0.122
	Medium	99 (40.7)	39 (39.4)	
	High	97 (39.9)	46 (47.4)	
*Type of place of residence*	Urban	114 (46.9)	53 (46.5)	0.086
	Rural	129 (53.1)	46 (35.7)	
*Partner’s occupation*	Agriculture	40 (16.5)	12 (30.0)	0.130
	Other	203 (83.5)	87 (42.9)	

In terms of prevalence within factor levels, Table 1 shows that only 18 (16.2%) of the 111 HIV- positive women (45.7% of the total sample) expressed a desire to have another child. On the contrary, among the 94 women (38.7%) who were between 25 and 34 years old, 50 (53.2%) wanted to have another child. Among the 117 women (48.1%) who were not in a union, 40.2% reported the desire for a child, while 43.5% of the 108 women who did not have a co-wife expressed the desire to have another child.

The prevalence of fertility desire was as low as 39.2% among HIV-positive women whose age at first sex was below 19 years, 39.0% in those who lived in only one sexual union, and 30.0% in those women whose partners were working in agriculture. On the contrary, the fertility desire was prevalent in 86.2% of women who had no living child, 81.6% of women aged 15 to 24 years old, 52.2% of women who were not using any contraceptive method, and 53.3% for those women who completed secondary or higher education. The results also indicated that, among the 114 women who were living in urban areas, 46.5% had a desire for more children, while 35.7% were in rural areas. The fertility desire was at 36.2% in the 196 HIV-positive women who were employed, while it was 59.6% in those who were not..

### Factors associated with fertility desire

Table 1 shows the results of tests of a statistical association between the woman’s fertility desire and each of the potential factors. At 5% level of significance, five factors showed a statistically significant association with fertility desire, namely, (i) the woman’s age, (ii) current contraceptive use, (iii) woman’s number of living children, (iv) woman’s current employment status, and (v) partner’s desire for children.

The analytical results in Table 2 show both the unadjusted odds ratios (OR) and adjusted odds ratios (AOR) as well as their corresponding 95% confidence intervals (CIs) for each of the factors included in the present study in order to assess the magnitude and direction of their effects on fertility desire of an HIV-positive woman. The four variables, which were individually associated with fertility desire, also turned out to be statistically significant factors associated with fertility desire among HIV-positive women in Rwanda in a multivariable analysis. Specifically, the results indicate that the most influential factors associated with fertility desire among HIV-positive women in Rwanda included the woman’s age, number of living children, employment status, and current contraceptive use. The *p*-value for Hosmer-Lemeshow goodness-of-fit test for the multivariable binary logistic model was 0.793 confirming that the model was generally a good fit at 5% level of significance. The overall percentage of correctly classified cases was 80.7%, indicating a high prediction power [40].

**Table 2:** Factors associated with fertility desire in HIV-positive women (*n* = 243)

Variables (Reference category)	Unadjusted Odds Ratio (95% CI)	Adjusted Odds Ratio (95% CI)
**Woman’s age (**15-24 years**)**	1	1
25–34 years	0.257 (0.103–0.641)	0. 387(0.119–1.259)
35–49 years	0.044 (0.017–0.114)	0. 051(0.013–0.204)*
**Marital status** (Living with partner)	1	1
Not in union	0.955 (0.572–1.595)	0.550 (0.075–4.008)
**Has co-wives** (No)	1	1
Other	0.813 (0.486–1.360)	0.348 (0.068–1.775)
**Sexually active** (No)	1	1
Yes	1.324 (0.793–2.212)	2.267 (0.725–7.082)
**Age at first sex** (Below 19 years)	1	1
19 years or above	1.140 (0.683–1.903)	1.713 (0.768–3.824)
**Number of sexual unions** (Once)	1	1
Other	1.186 (0.707–1.991)	1.037 (0.448–2.398)
**Ideal number of children** (At most 2)	1	1
3 or above	0.703 (0.406–1.219)	2.122 (0.912–4.936)
**Number of living children** (None)	1	1
1–2	0.204 (0.066–0.628)	0.611 (0.153–2.444)
3 or above	0.024 (0.007–0.080)	0.177 (0.037–0.837)*
**Partner’s desire for children** (Wants the same)	1	1
Other	1.380 (0.800–2.381)	3.752 (1.203–11.702)*
**Current contraceptive use** (No)	1	1
Yes	0.215 (0.115–0.403)	0.146(0.057–0.375)*
**Woman’s Religion** (Catholic)	1	
Protestant	1.371 (0.766–2.452)	1.268 (0.532–3.022)
Other	1.201 (0.584–2.470)	1.198 (0.416–3.446)
**Currently employed** (No)	1	1
Yes	0.385 (0.201–0.739)	0.298 (0.113–0.782)*
**Economic status** (Low)	1	1
Medium	1.532 (0.728–3.224)	2.447 (0.833–7.193)
High	2.126 (1.013–4.462)	3.537 (0.843–14.845)
**Type of place of residence** (Urban)	1	1
Rural	0.638 (0.381–1.068)	1.196 (0.427–3.352)
**Woman’s education (No education)**	1	1
At most primary	1.138 (0.570–2.272)	0.571 (0.195–1.678)
Secondary or higher	2.071 (0.889–4.827)	1.137 (0.287–4.502)
**Partner’s occupation** (Agriculture)	1	1
Other	1.750 (0.842–3.636)	1.171 (0.368–3.726)
Constant	–	4.803
Hosmer-Lemeshow goodness-of-fit test *p*-value	–	0.793
Prediction power (% of correctly classified)	–	80.7%

*statistically significant effect with respect to the reference category

The results in Table 2 show that AOR were higher than unadjusted odds ratios for three of the five significant factors, namely the number of woman’s living children, the partner’s desire for children, and the woman’s age. The presence of the other factors in the model amplified their respective effects. The adjusted odds ratio decreased significantly with increase in the woman’s age. In particular, the adjusted odds ratio of desire to have another child was 0.051 (95% CI: 0.013–0.204) for woman’s age 35–49 years, which indicates that the latter were associated with more than 90% smaller odds of desire to have another child than those who were 15–24 years old. Although, women aged 25 to 34 years were associated with 61.3% lesser odds of desire to have another child (AOR = 0.387, 95% CI: 0.119–1.259) than those who were 15–24 years old, this difference in fertility desire was not statistically significant.

Similarly, HIV-positive women who had 3 or more living children were significantly associated with 82.3% lesser odds of desire to have another child (AOR = 0.177, 95% CI: 0.037–0.837) than those who did not have any living child. There was also important difference in fertility desire, but not statistically significant, between women with 1–2 living children (AOR = 0.611, 95% CI: 0.153–2.444) and those who did not have any living child.

In addition, the adjusted odds ratio was 0.298 (95% CI: 0.113–0.782) for employed women, representing more than 70% lesser odds of desire for more children for HIV-positive women who were employed compared to those who were not employed. Further, HIV-positive women who were using contraceptive were slightly more than 85% less likely to desire having another child (AOR = 0.146, 95% CI: 0.057–0.375) compared to those women who were not using any contraceptive method. The partner’s desire for children (AOR = 3.752, 95% CI: 1.203–11.702) turned out to be a significantly influential factor associated with fertility desire.

Although, their effects were not statistically significant, the ideal number of children above 2, being sexually active, and living in household with high or medium economic status were associated with increase in fertility desire among HIV-positive women.

## Discussion

This study aimed to assess fertility desire among HIV- positive women and its determinants in Rwanda, where research-based evidence is still needed to inform the planning of health care service provision to HIV-positive women. The present study is based on the 2015 RDHS, which is the nationally representative survey in Rwanda that provides data for HIV prevalence estimation with a wide range of socioeconomic, demographic, household’s environment, health and behavioural characteristics.

The results showed that fertility desire among women living with HIV/AIDS (WLHA) in Rwanda is 40.7%. Although this is less than the prevalence in the general women population (49%) [8], it constitutes an important component of the national prevalence rate of fertility desire among the women population in Rwanda. Almost the same prevalence of fertility desire (40.3%) was estimated among HIV-positive women in a study conducted in referral hospitals in Northwest Ethiopia in 2017 [22].

Different studies led to mixed results dominated by an increasing desire for children in PLHA. For example, a study conducted in Rwanda did not find any significant difference in fertility between HIV-positive and HIV-negative women [41], while the results from studies conducted in Ethiopia, Kenya and South Africa among PLHA reported prevalence of desire for more children that was considerably lower (33.4, 34 and 44% respectively) than in the general population or HIV-negative counterpart [4, 18]. On the contrary, the prevalence of the desire to have more children among HIV-positive women was as high as 54.6% among HIV-positive women of reproductive age in Addis Ababa, Ethiopia [6]. It was also reported to be higher in HIV-positive men than in HIV-positive women [30].

We found that the woman’s number of living children was a significant determinant of the desire to have more children among WLHA in Rwanda. The positive effect of having few living children on fertility desire and intention has been reported in several studies [4, 22, 23, 30, 31, 42]. In this study, women with no living child were associated with greater odds of the desire to have a child than any other woman. Studies show that, in some communities, social pressure significantly influences a woman’s desire to have a child as the only way to experience motherhood or qualify as a ‘woman’ [4, 25]. In the particular context of Rwanda, a family that has a child is considered blessed.

This study showed that the woman’s age was negatively associated with her desire to have another child. In particular, women who were between 35 and 49 years old were associated with a lesser desire of having more children than any other woman. These women are in the advanced maternal age and most of them might be closer to their desired parity [43]. Similarly, several studies reported a negative relationship between a woman’s age and her fertility desire, especially among HIV-positive women [4, 22, 23].

Further, the employment status of an HIV-positive woman had a statistically significant association with her desire to have another child in Rwanda. Women who were employed were associated with lesser odds of desire to have another child than those who were unemployed. Some previous studies did not detect any significant effect of employment status or working time on a woman’s fertility [44] while others found it [26–28]. The data at hand revealed that women who were employed were associated with a low likelihood of a desire to have a child in the future. In Rwanda, the woman’s employment status is often dependent on her level of education and constitutes an important explanation of her income and living standard as well as her use of health care services. Thus, the link between employment status and fertility desire in Rwanda needs further investigation.

Finally, the use of contraceptives was a statistically significant predictor of women fertility desire after controlling for other factors’ effects. There was a very low likelihood of desire to have another child among HIV-positive women who were using a modern contraception method. This result corroborates several other study findings according to which women who use contraceptive methods to control their fertility are associated with a low probability of desire to have more children [42, 43, 45, 46].

In general, demographic and socio-economic factors are the most influential drivers of HIV-positive woman’s fertility desire in Rwanda. The results point to an increased desire for more children among HIV-positive women that mainly lead to changes in their sexual and reproductive behaviours [5, 20, 41]. Early initiation and successful scale-up of antiretroviral therapy played a potential role in many counties including Rwanda [15–19].

The results suggest that the desire for children in HIV-positive women in Rwanda was rooted in the culture and traditional consideration of the high social value of having own child [4, 25]. The key factors associated with the desire for children are dominated by demographic factors, which can be difficult for the health system to influence significantly without harm to reproductive rights. The results imply that there will be, among other things, a sustained high demand for services of PMTCT of HIV, and an increased utilization of the antenatal and postnatal care services as pregnant HIV-positive women will want to ensure a positive pregnancy outcome [47] . Further, the integration of HIV testing into antenatal care services will remain an important means for HIV infection screening in pregnant women [48]. The more the partner desires to have another child, the more the woman will desire to satisfy her partner’s need. In the particular context of Rwanda, the partner’s expressed need for a child, makes the woman feel appreciated and she considers a child as a lifelong gift to her husband and extended family. Research has shown that women can even experience pressure to have children even from the family or community members [4.25].

This study presents some limitations. First, this study used cross-sectional survey data and thus it cannot establish causality between the outcome and the predictors but associations. The data did not also have information on women’s history of antiretroviral therapy. However, in the context of high coverage of HIV/AIDS related services [1, 15], these limitations cannot affect the quality of the data and methods we used to analyse them. Thus, the results remain valid and can be used as evidence for policy formulation and design of optimal health programs for improved maternal and child health services in the context of Rwanda and similar settings.

## Conclusion

The present study contributes to the understanding of the ongoing changes in fertility desire among WLHA in Rwanda and other developing countries that realized significant improvement in the life expectancy of HIV/AIDS patients. The study revealed that the high fertility desire among HIV-positive women in Rwanda is largely dependent on demographic and socioeconomic factors. The results imply that the health care system should be prepared to intensify the required services for the PMTCT of HIV, the delivery of maternal and child health care services, and the support to WLHA in planning their fertility. Interventions among WLHA in Rwanda should target young women with fewer living children and with a particular focus on meeting their contraceptive needs.

## Data Availability

The datasets supporting the conclusions of this article (Individual Women’s data and HIV test data from the 2015 RDHS) are freely available upon request, in the DHS program repository at: https://dhsprogram.com/data/dataset/Rwanda_Standard-HS_2015.cfm?flag=0

## Abbreviations

AOR: Adjusted odds ratio
CI: Confidence interval
HIV/AIDS: Human immunodeficiency virus/ Acquired immunodeficiency syndrome
PLHA: People living with HIV/AIDS
RDHS: Rwanda demographic and health survey
PMTCT: Prevention of mother-to-child transmission
UNAIDS: Joint United Nations Programme on HIV/AIDS
WLHA: Women Living with HIV/AIDS
